# Driven by fatigue and fear: the push-pull mechanism of information overload on reflective smartphone disengagement

**DOI:** 10.1080/00049530.2026.2705452

**Published:** 2026-07-27

**Authors:** Ping Han, Yiwei Wu, Lin Cai

**Affiliations:** a School of Information and Communication, Communication University of China, Beijing, China; b School of Marxism, Sichuan Institute of Industrial Technology, Deyang, China; c School of Chinese National Community, Southwest Minzu University, Chengdu, China

**Keywords:** Social media fatigue, fear of missing out, protection motivation theory, self-efficacy

## Abstract

**Objective:**

Information overload can exhaust cognitive and psychological resources, prompting individuals to adopt reflective smartphone disengagement as a protective strategy. Yet, users often experience fatigue while fearing missing out on important updates, trapping them in a psychological dilemma between connection and disconnection. Grounded in protection motivation theory, this study investigates the dual-pathway mechanism linking information overload to reflective smartphone disengagement, specifically testing social media fatigue and fear of missing out as mediators, and self-efficacy as a moderator.

**Methods:**

A cross-sectional survey was conducted among 2,508 participants in Sichuan Province, China. SPSS 26.0 was used for descriptive statistics, and Mplus 8.3 for mediation and moderation analyses.

**Results:**

The findings demonstrated a significant positive correlation between information overload and reflective smartphone disengagement. Social media fatigue significantly mediated this relationship. The positive associations linking information overload, social media fatigue, and reflective smartphone disengagement were weaker at higher levels of self-efficacy.

**Conclusion:**

Facing information overload, students experience social media fatigue and fear of missing out, which reduces their likelihood of reflective disengagement. Moreover, protective behaviours relate to personal beliefs. Students with lower self-efficacy are highly motivated to eliminate threats directly, frequently adopting reflective smartphone disengagement as a protective strategy.

## KEY POINTS

What is already known about this topic:

1. Information overload has negative effects, often associated with the adoption of digital disconnection as a protective strategy.
2. Protection motivation theory provides a robust framework for understanding how individuals appraise threats and their coping capabilities.
3. Navigating digital information overload is frequently linked to a psychological paradox, characterised concurrently by social media fatigue and the fear of missing out. People are caught in a dilemma between staying connected and disconnection.

What this topic adds:

1. Reflective smartphone disengagement is not merely a direct correlate of information overload; rather, it reflects a complex psychological negotiation between the “push” of fatigue and the “pull” of the fear of missing out.
2. Self-efficacy functions as a vital internal resource, corresponding with a decreased reliance on rigid disconnection when managing digital threat.
3. Integrating reflective smartphone disengagement into the protection motivation theory framework provides a novel theoretical lens, conceptualising smartphone disengagement as a protective coping mechanism.

## Introduction

While digital technologies dismantle the barriers of time and space to facilitate social interaction, a large amount of information often exceeds an individual’s processing capacity, leading to information overload (Misra & Stokols, 2012; Tandoc & Kim, 2023). The rapid proliferation of digital media compels users to rapidly filter and evaluate vast quantities of varied content (Ye et al., 2023). Simultaneously, the necessity to navigate multiple platforms drives them towards chronic multitasking (Madianou & Miller, 2013; Sheng et al., 2023). Research indicates that 49% of university students struggle to manage and process such extensive information (Neiterman & Zaza, 2019; Zaremohzzabieh et al., 2024). For university students, their cognitive faculties are still maturing, experiencing information overload makes these individuals particularly vulnerable to cognitive biases and failures, thereby impairing effective task execution (Neave et al., 2019; Soroya et al., 2021). Beyond compromised performance, the resulting behavioural and psychological burdens often precipitate severe health issues (Misra & Stokols, 2012; Swar et al., 2017).

According to the protection motivation theory (Rogers, 1975), individuals who perceive discomfort and psychological threats from information overload typically engage in protective behaviours. The most immediate and straightforward action involves deliberately distancing oneself from smartphones, a phenomenon conceptualised as reflective smartphone disengagement (Matthes et al., 2022). By serving as a defensive mechanism against the detrimental impacts of screen time, reflective smartphone disengagement enables students to redirect their cognitive resources towards offline activities. Given their status as digital natives, university students consume vast amounts of digital content but frequently struggle with information management (Neiterman & Zaza, 2019), rendering them exceptionally susceptible to information overload. Consequently, elucidating the drivers behind their protective actions is crucial for mitigating cognitive failure and psychological harm.

Nevertheless, in a deeply connected society, complete digital disconnection is often unrealistic (Syvertsen & Enli, 2020). Although students may feel tired and overwhelmed by information overload, they may also fear missing important updates. This creates a psychological tension between the wish to disconnect and the need to remain connected (L. Li et al., 2022). As a result, people do not always respond to information overload with immediate protective action. Instead, the move from information overload to reflective smartphone disengagement may depend on how they interpret the situation and evaluate their coping options. Drawing on protection motivation theory, this study examines the indirect links between information overload and reflective smartphone disengagement. In particular, it focuses on the mediating roles of social media fatigue and fear of missing out, as well as the moderating role of self-efficacy.

## Literature review

### Theoretical basis

Protection motivation theory (Rogers, 1975) posits that individuals cognitively appraise perceived threats, generate protective motivations, and employ strategies to mitigate harm. As outlined by Rogers et al. (1983), the model comprises three stages: exposure to a situation, cognitive appraisal and coping responses. Serving as the theoretical core, cognitive appraisal connects the initial stimulus to protective behaviours through both threat and coping evaluations. Drawing on this framework, the present research conceptualises information overload as the environmental stimulus and reflective smartphone disengagement as the protective behaviour.

### Reflective disconnection: reflective smartphone disengagement and information overload

Matthes et al. (2022) introduced reflective smartphone disengagement, defining it as a deliberate effort to control the smartphone use (Baumeister, 2007). Particular emphasis is placed on the prefix “reflective”. Grounded in self-regulation theory and the impulsive nature of human cognition (Baumeister, 2007; Hofmann et al., 2009), reflective smartphone disengagement functions as a protective behaviour enacted to safeguard well-being against perceived threats. Moreover, rather than a passive reaction, it constitutes a proactive practice requiring users to navigate complex psychological conflicts. Reflective smartphone disengagement entails intentionally establishing personal rules for device access, meaning it is neither the direct opposite of impulsive use nor a complete rejection of technology. Unlike general non-use, which imposes broad limitations, reflective smartphone disengagement involves context-specific restrictions (Vanden Abeele, 2020). Ultimately, adopted following careful psychological deliberation, reflective smartphone disengagement is a protective behaviour, aiming to seek an optimal balance between connection and disconnection (Matthes et al., 2022). This concept also reflects the complex psychology of users in contemporary digital practice.

Information overload was defined as a situation where the volume of information exceeds a person’s ability to process it (Miller, 1956; Tandoc & Kim, 2023). Because smartphones constantly push notifications, news and social updates, the endless stream of content demands constant attention and drains cognitive resources. As a result, users often feel a loss of control and high levels of anxiety (Lee et al., 2016). Viewed through the lens of protection motivation theory, information overload acts as a severe environmental threat. When people realise that they cannot effectively process such massive amounts of information, they experience deep cognitive fatigue, prompting them to seek strategy to protect their mental well-being (Pang & Ruan, 2023).

While stepping away from the device seems like a logical defence, smartphones are too deeply woven into daily work and social life to abandon entirely. Because total disconnection is unrealistic for most people (Syvertsen & Enli, 2020), users must instead rely on reflective strategies. By evaluating the immediate threat, they can intentionally set healthy boundaries. Past studies show that people actively use methods like content bypassing and temporary breaks to avoid the threat of information overload (Volk et al., 2025). Among these approaches, reflective smartphone disengagement serves as a vital self-regulatory tool. By enabling users to block out excessive noise while keeping necessary digital connections (Matthes et al., 2023), such behaviour functions as an adaptive protective strategy to avoid the threat of information overload. Based on these analyses, the research proposed the hypothesis:

H1: Information overload is positively associated with reflective smartphone disengagement.

### The interplay within protection motivation theory: social media fatigue and fear of missing out

Because reflective smartphone disengagement relies on cognitive processes, understanding the complex and often conflicting psychological responses to information overload is essential. Past studies show that information overload is closely associated with two specific psychological states: social media fatigue and fear of missing out (Dhir et al., 2018). Within the protection motivation theory, these states act as opposing forces that relate to disconnect.

Social media fatigue is a negative emotional state characterised by exhaustion and annoyance (Lee et al., 2016; Ou et al., 2023). From the perspective of threat appraisal, such fatigue reflects the psychological cost and perceived severity of staying online during periods of information overload. For users facing information overload, the heavy mental effort required to process low-quality content is strongly correlated with feeling fatigue (Sheng et al., 2023). Consequently, higher levels of social media fatigue correspond with a stronger motivation to conserve mental energy, a state highly associated with protective strategies like reflective smartphone disengagement (Ou et al., 2023).

Conversely, fear of missing out describes the anxiety individuals experience over potentially missing important information or social interactions (Tanhan et al., 2022). Despite the burden of information overload, the perpetual updates of information fuel the apprehension that overlooking any piece of content may lead to social disadvantages (Chiu, 2021). And being confronted with information overload is associated with depleted mental resources and a diminished ability to filter effectively, a state that correlates with intensified fear of missing out (Ye et al., 2023). To alleviate the unease of social exclusion and stay aligned with peers, people experiencing such anxiety frequently use their devices to monitor real-time updates. For those relying heavily on social media to stay informed, fear of missing out corresponds with a higher perceived value of connectivity and is strongly associated with ongoing smartphone use (L. Li et al., 2022; Yin et al., 2021). Therefore, individuals experiencing higher levels of fear of missing out are less likely to engage in reflective smartphone disengagement.

When confronting information overload, individuals do not solely perceive social media fatigue or fear of missing out. Rather, they grapple with a conflicting interplay of both emotions, undergoing a complex process of perception and appraisal before deciding whether to adopt reflective smartphone disengagement as a protective strategy. Within protection motivation theory, these two psychological states represent countervailing forces in the threat appraisal phase. Individuals find themselves caught between the dread of missing important information and the sense of exhaustion induced by a large quantity of complex information. Threat appraisal is the first step, evaluating the perceived severity of threat, fear, etc., followed by a balancing of these factors (Solomon et al., 1989). Prior research indicates that higher perceived threat increases the likelihood of adopting protective measure (Inkeles & Smith, 1974). However, other factors can counteract the perceived threat (Floyd et al., 2000). Prevention motivation tends to be lower when perceived fear of missing out outweigh the perceived threats (social media fatigue). Applying protection motivation theory, the ultimate appraisal of information overload represents an outcome of balancing social media fatigue and fear of missing out. When social media fatigue takes the lead, information overload needs to be avoided, thereby reinforcing its preventive motivation. When fear of missing out dominates, individuals tend not to take protective actions and remain connected. Based on these associations, the following hypotheses are proposed:

H2: Information overload is positively associated with social media fatigue.

H3: Information overload is positively associated with fear of missing out.

H4: Social media fatigue is positively associated with reflective smartphone disengagement.

H5: Fear of missing out is negatively associated with reflective smartphone disengagement.

H6: Social media fatigue mediates the relationship between information overload and reflective smartphone disengagement, yielding a positive indirect effect.

H7: Fear of missing out mediates the relationship between information overload and reflective smartphone disengagement, yielding a negative indirect effect.

### The moderating role of self-efficacy

According to protection motivation theory, once an individual completes the threat appraisal, they enter the stage of coping appraisal, evaluating their ability to execute preventive behaviours (Rogers, 1975). As a core component of coping appraisal, self-efficacy is defined as a person’s stable belief in their capacity to mobilise the motivation and cognitive resources needed to meet environmental demands (Bhati & Sethy, 2022). High self-efficacy often correlates with active risk management and a greater willingness to address environmental challenges (Inkeles & Smith, 1974). Because self-regulation is deeply rooted in these beliefs, high self-efficacy strongly correlates with better control over health behaviours, the active use of preventive interventions and successful risk avoidance (Rasool et al., 2024). Within the current research, reflective smartphone disengagement represents a behavioural strategy used to avoid threats (Matthes et al., 2023). Because executing reflective smartphone disengagement requires confidence, individuals must simultaneously modify ingrained habits and manage the potential social consequences of disconnecting (Matthes et al., 2022).

Specifically, when experiencing information overload or social media fatigue, individuals with high self-efficacy are more likely to view these conditions as actionable signals rather than overwhelming burdens. Self-efficacy strengthens individuals’ tendency to translate perceived risks into protective actions (Świątek et al., 2023). Additionally, their confidence enables them to implement reflective disengagement as a deliberate protective strategy, trusting that they can handle the temporary disconnection. In contrast, individuals lacking this internal resource often doubt their ability to handle social isolation or sudden information influxes, frequently struggling to manage their device connectivity (Qin et al., 2024). Furthermore, fear of missing out is closely tied to difficulties in regulating cognitive demands (Przybylski et al., 2013). Stronger self-regulatory resources typically align with greater independence, autonomy and confidence in maintaining healthy social connections (Morelli et al., 2023). When experiencing fear of missing out, individuals with high self-efficacy can rely on their internal capabilities to effectively manage information interference, whereas low self-efficacy individuals may feel trapped by the anxiety of missing out. Therefore, self-efficacy should amplify the relationship between them and disengagement.

Therefore, students’ level of self-efficacy is strongly associated with their general attitudes and behavioural strategies. Consequently, we anticipate that high self-efficacy will correspond with stronger positive associations of information overload, social media fatigue, and fear of missing out with reflective smartphone disengagement.

H8: Self-efficacy positively moderates the respective associations of (a) information overload, (b) social media fatigue, and (c) fear of missing out with reflective smartphone disengagement. Specifically, this paper supposes that these positive relationships are stronger at higher levels of self-efficacy.

**Figure 1.**
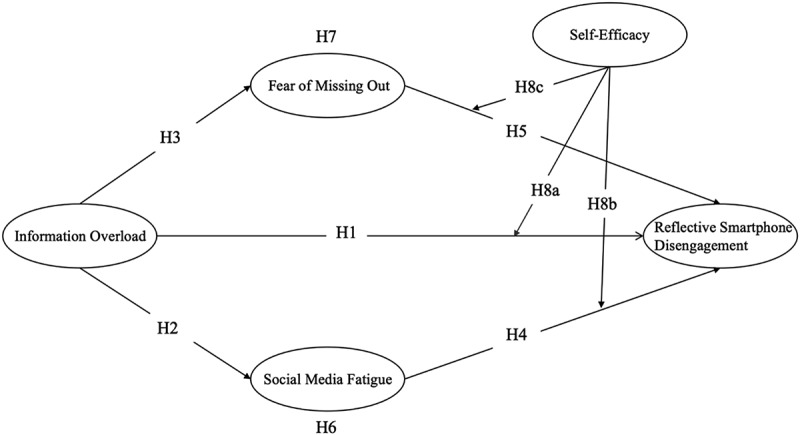
Hypothetical model.

Note. H6 and H7 denote the mediation hypotheses; H8a, H8b, and H8c denote the moderation hypotheses of self-efficacy.

Figure 1 presents the conceptual model and hypotheses of the current study.

## Material and methods

### Participants

Conducted in Sichuan Province, China, between May and June 2024, this study targeted university students aged 18 to 24, an age bracket aligning with the standard from the National Centre for Education Statistics (National Centre for Education Statistics, 2023). University students are a highly active group on social media, utilising smartphones for social network and academic. Therefore, they are more likely to suffer from information overload (Neiterman & Zaza, 2019; Zaremohzzabieh et al., 2024). Following standardised ethics training for all investigators, university teachers organised students to complete the survey uniformly via the Wenjuanxing platform. The first page explicitly detailed the research purpose and confirmed the absence of any conflicts of interest. After reading this information, participants voluntarily provided informed consent. To maintain objectivity, survey administrators offered no guidance or suggestions during completion.

To prevent data bias and ensure fair representation across grade levels, a quota sampling approach was employed. The inclusion criteria required participants to: (1) hold Chinese nationality; (2) voluntarily consent to participate; (3) independently complete and understand the survey items; and (4) not be simultaneously involved in similar research. During the subsequent data review, responses were excluded if they: (1) were completed unusually quickly; (2) showed uniform answering patterns (e.g., selecting the exact same option throughout); or (3) failure to pass lie detection tests (e.g., we will require a particular item to be answered “B” to check whether respondents have read the questions properly). Ultimately, the final sample comprised 2,508 valid responses.

### Ethical consideration

All methods in our study complied with the Declaration of Helsinki. This study has been approved by the Ethics Committee of the Psychology and Behavioural Science Research Centre of the Philosophical and Social Sciences Association of Deyang, Sichuan Province, China, under the approval number: DYXLYXW2024-02. Prior to participation, all respondents provided informed consent, were assured of the confidentiality of their responses, and were free to withdraw at any time without penalty. No personally identifiable information was collected, and data were analysed only in aggregate form.

### Measurements

#### Reflective Smartphone Disengagement (RSD)

Reflective smartphone disengagement was assessed using the scale originally developed by Matthes et al. (2022) This 6-item instrument measures the extent of an individual’s deliberate efforts to reduce or avoid smartphone use in specific contexts and has demonstrated high test-retest reliability and discriminant validity. The scale captures individuals’ deliberate restrictions on smartphone use in specific situations, and rates their agreement on a five-point Likert scale (1 = Strongly Disagree, 5 = Strongly Agree). A representative item is, “At certain times of the day (e.g., during meals), I don’t want to use my smartphone”. Higher scores indicate a greater tendency for reflective smartphone disengagement. In this study, the Cronbach’s alpha coefficient of the scale was 0.841, and the McDonald’s omega coefficient was 0.843.

#### Information Overload (IO)

Information overload was measured using the three-item unidimensional scale validated by Whelan et al. (2020). This scale focuses on perceptions of information and communication overload. Respondents indicated their level of agreement with the statements on a seven-point Likert scale (1 = Strongly Disagree, 7 = Strongly Agree), with higher scores reflecting a greater perceived degree of information overload from social media. A representative item is, “I am often distracted by the excessive information on social media”. In this study, the Cronbach’s alpha coefficient of the scale was 0.893, and the McDonald’s omega coefficient was 0.893.

#### Social Media Fatigue (SMF)

Social media fatigue was measured using a four-item unidimensional scale adapted from Lee et al. (2016). The Chinese version of the scale, translated and simplified by Huang (2020), was employed and has established reliability. Respondents rated their experience of fatigue related to social media use on a five-point Likert scale (1 = Strongly Disagree, 5 = Strongly Agree). A representative item is, “I find it hard to relax after continuous use of social media”. Higher scores indicate greater social media fatigue. In this study, the Cronbach’s alpha coefficient of the scale was 0.883, and the McDonald’s omega coefficient was 0.884.

#### Fear of Missing Out (FoMO)

Fear of missing out was assessed using the scale developed by Przybylski et al. (2013). The Chinese adaptation by Q. Li et al. (2019), which has been validated for use with Chinese university students, was adopted. This 8-item, two-dimensional scale requires respondents to rate the frequency of their concerns about missing out on information and social interactions when using smartphones. Responses are captured on a five-point frequency scale (1 = Never, 5 = Always), where higher scores indicate greater digital pressure associated with fear of missing out. A representative item is, “I fear others have more rewarding experiences than me”. In this study, the Cronbach’s alpha coefficient of the scale was 0.783, and the McDonald’s omega coefficient was 0.727.

#### Self-Efficacy (SE)

Self-efficacy was measured using a three-item unidimensional scale sourced from the Psychology and Behaviour Investigation of Chinese Residents (PBICR) (Wang et al., 2022). This scale assesses an individual’s confidence in overcoming difficulties. Responses were recorded on a five-point Likert scale (1 = Strongly Disagree, 5 = Strongly Agree), with higher scores representing stronger self-efficacy in facing challenges. A representative item is, “When faced with difficult tasks, I am convinced that I can complete them”. In this study, the Cronbach’s alpha coefficient of the scale was 0.953, and the McDonald’s omega coefficient was 0.953.

#### Control variable

Given that all participants were university students, the sample was relatively homogeneous in age, educational setting, and media use context, which is typical of student samples (Peterson, 2001; Sears, 1986). We therefore included gender as a basic control variable rather than adding multiple demographic controls without clear theoretical relevance (Becker et al., 2016). However, we acknowledge that controlling only for gender is limited and does not account for other potentially relevant individual differences. Gender was coded as 1 = male and 2 = female.

### Data processing

Following data screening, statistical analyses were conducted using SPSS 26.0 and Mplus 8.3. Initially, descriptive statistics and bivariate correlations were computed to examine variable distributions, identify potential outliers, and assess preliminary relationships. Multicollinearity tests revealed that all variance inflation factor (VIF) values remained below 5, indicating the absence of significant multicollinearity within the dataset (Menard, 2021).

Confirmatory factor analysis (CFA) was performed to evaluate the measurement model. The results demonstrated a good model fit, with RMSEA (Root Mean Square Error of Approximation) = 0.057 (<0.08), TLI (Tucker-Lewis Index) = 0.952 (>0.90), and CFI (Comparative Fit Index) = 0.961 (>0.90), satisfying the fit criteria proposed by Hu and Bentler (1999). We further assessed common method bias using the unmeasured latent method construct (ULMC) approach (Dan-Dan & Zhong-Lin, 2020). After introducing the latent method factor, RMSEA = 0.053, TLI = 0.959, and CFI = 0.967. The resulting index variations (ΔRMSEA < 0.015, ΔCFI < 0.01, and ΔTLI < 0.01) fell well within the prescribed thresholds (Podsakoff et al., 2003), confirming the absence of significant common method bias.

Structural equation modelling (SEM) with maximum likelihood estimation was conducted in Mplus 8.3 to test the hypothesised paths. A baseline mediation model, excluding the moderator, was first constructed to examine the indirect associations involving fear of missing out and social media fatigue. Subsequently, self-efficacy was introduced into the structural model to test the moderated mediation relationships. To rigorously assess these complex models, a bias-corrected bootstrapping method with 5,000 iterations was utilised. This hierarchical approach clarified how self-efficacy moderated the overall structural paths. To comprehensively report all structural variations, conditional associations were calculated at three levels of the moderator: the mean (M), one standard deviation below the mean (M – SD), and one standard deviation above the mean (M + SD).

## Results

### Descriptive statistics and correlations

The sample comprised 1,043 males (41.6%) and 1,465 females (58.4%) participants. Table 1 details the means, standard deviations, and the bivariate correlations. As expected, information overload was positively correlated with both reflective smartphone disengagement (*r* = 0.107, *p* < 0.001) and social media fatigue (*r* = 0.536, *p* < 0.001). Furthermore, social media fatigue positively correlated with reflective smartphone disengagement (*r* = 0.282, *p* < 0.001). These initial findings provided preliminary support for the proposed associations, justifying further evaluation of the hypothesised structural model.

**Table 1.** Descriptive statistics and correlations among key variables.

Variable	Mean	SD	1. IO	2. RSD	3. SMF	4. FoMO	5. SE
1. IO	11.645	3.743	1.000				
2. RSD	18.812	4.436	0.107***	1.000			
3. SMF	11.940	3.393	0.536***	0.282***	1.000		
4. FoMO	21.984	5.326	0.327***	0.038	0.271***	1.000	
5. SE	10.202	2.803	0.051*	0.331***	0.104***	−0.032	1.000

Note. *p > 0.050, **p < 0.010, ***p < 0.001. *M* = Mean; SD = Standard Deviation; IO = Information Overload; RSD = Reflective Smartphone Disengagement; FoMO = Fear of Missing Out; SMF = Social Media Fatigue; SE = Self-Efficacy.

### Mediation model evaluation

Prior to model evaluation, all variables were mean-centred. As Table 2 details, the model fit demonstrated acceptable results: CFI = 0.965, TLI = 0.957, RMSEA = 0.054. The results indicated that information overload positively correlated with both fear of missing out (β = 0.194, 95% CI [0.169, 0.221]) and social media fatigue (β = 0.439, 95% CI [0.402, 0.476]). Therefore, H2 and H3 were supported. Furthermore, both social media fatigue (β = 0.348, 95% CI [0.280, 0.416]) and information overload (β = 0.073, 95% CI [0.022, 0.122]) demonstrated significant positive correlations with reflective smartphone disengagement. However, fear of missing out didn’t show a significant association with reflective smartphone disengagement. These supported H1 and H4, but H5 was not supported. Regarding the mediation analysis, the results revealed that social media fatigue positively significantly mediated the relationship between information overload and reflective smartphone disengagement (β = 0.152, 95% CI [0.121, 0.186]). H6 was supported. Unexpectedly, the specific indirect association through fear of missing out was not statistically significant. H7 was not supported.

**Table 2.** Test of the mediation model.

		β	se	*p*	LLCI	ULCI
Direct Effect	IO→FoMO	0.194	0.014	<0.001	0.169	0.221
	IO→SMF	0.439	0.019	<0.001	0.402	0.476
	FoMO→RSD	−0.033	0.038	0.392	−0.108	0.039
	SMF→RSD	0.348	0.035	<0.001	0.280	0.416
	IO→RSD	0.073	0.026	0.005	0.022	0.122
Indirect Effect	IO→FoMO→RSD	−0.006	0.007	0.391	−0.021	0.008
	IO→SMF→RSD	0.152	0.017	<0.001	0.121	0.186

Note. IO = Information Overload; RSD = Reflective Smartphone Disengagement; FoMO = Fear of Missing Out; SMF = Social Media Fatigue; SE = Self-Efficacy.

### Moderation analysis

Upon incorporating self-efficacy as a moderating variable (Table 3), the final structural model demonstrated a good fit: CFI = 0.963, TLI = 0.958, RMSEA = 0.044, and SRMR = 0.042. The fundamental associations among the primary variables exhibited minor numerical variations but remained statistically significant. The analysis revealed significant negative interaction effects. Specifically, self-efficacy negatively moderated the association between information overload and reflective smartphone disengagement (β = −0.030, 95% CI [−0.045, −0.015]). To clarify this mechanism, conditional associations were evaluated across varying levels of self-efficacy (Table 4). The results indicated that as self-efficacy increased, the positive relationship between information overload and reflective smartphone disengagement progressively weakened. Similarly, self-efficacy negatively moderated the relationship between social media fatigue and reflective smartphone disengagement (β = −0.023, 95% CI [−0.044, −0.002]). Unexpectedly, the results were contrary to H8a and H8b. As detailed in Table 4, although this positive association remained significant across all levels, its magnitude steadily diminished at higher levels of self-efficacy. However, self-efficacy didn’t play a significant moderating role between fear of missing out and reflective smartphone disengagement (β = −0.008, 95% CI [−0.034, 0.019]). H8c was not supported.

**Table 3.** Moderating effect.

Outcome	Predictor	β	se	*P*	95% CI	
					LLCI	ULCI
FoMO	IO	0.182	0.010	<0.001	0.162	0.203
SMF	IO	0.338	0.012	<0.001	0.314	0.362
RSD	IO	0.053	0.026	0.042	0.002	0.104
	FoMO	−0.051	0.041	0.223	−0.132	0.031
	SMF	0.470	0.037	<0.001	0.398	0.543
	SE	0.404	0.031	<0.001	0.344	0.465
	IO × SE	−0.030	0.007	<0.001	−0.045	−0.015
	FoMO × SE	−0.008	0.014	0.580	−0.034	0.019
	SMF × SE	−0.023	0.011	0.033	−0.044	−0.002

Note. *p < 0.050, **p < 0.010, ***p < 0.001. IO = Information Overload; RSD = Reflective Smartphone Disengagement; FoMO = Fear of Missing Out; SMF = Social Media Fatigue; SE = Self-Efficacy.

**Table 4.** Path coefficients across varying levels of the moderator.

Path	values of the moderator	Path Coefficient and Confidence Interval			
		β	SE	LLCI	ULCI
IO→RSD	M – 1SD	0.137	0.032	0.075	0.199
	M	0.053	0.026	0.002	0.104
	M + 1SD	0.031	0.036	−0.04	0.102
SMF→RSD	M – 1SD	0.534	0.05	0.436	0.632
	M	0.470	0.037	0.398	0.543
	M + 1SD	0.406	0.045	0.318	0.495

Note. IO = Information Overload; RSD = Reflective Smartphone Disengagement; FoMO = Fear of Missing Out; SMF = Social Media Fatigue; SE = Self-Efficacy.

## Discussion

In digital era, information technology determines the important challenges related to the relationship between well-being and the digital world (Lin et al., 2011). Improper strategy to cope with information overload will be the cause of damage to students’ psychological health (Misra & Stokols, 2012; Swar et al., 2017). The present study aimed to explore the underlying associations linking information overload to reflective smartphone disengagement in the digital environment. Guided by protection motivation theory, this research examined a dual-mediation framework involving social media fatigue and fear of missing out, alongside the moderating role of self-efficacy. The findings reveal that social media fatigue plays a significantly positive mediating role in the association between information overload and reflective smartphone disengagement. Self-efficacy plays a moderating role between information overload and reflective smartphone disengagement, social media fatigue and reflective smartphone disengagement, the moderating effects were negative rather than positive.

### The contradictory psychology of threat appraisal

Viewed through the lens of threat appraisal, information overload may be positively associated with two competing psychological responses: social media fatigue and fear of missing out. Students facing information overload may therefore experience a genuine psychological paradox. On one hand, persistent exposure to large volumes of digital information may coincide with the depletion of cognitive and emotional resources, which is consistent with social media fatigue. On the other hand, the same environment may also be associated with stronger concern about missing socially or informationally relevant updates, corresponding to higher levels of fear of missing out. Prior work has conceptualised fear of missing out as a persistent apprehension that others may be having rewarding experiences without oneself, accompanied by a desire to stay continually connected with what others are doing (Przybylski et al., 2013). In this sense, information overload may not only be linked to exhaustion among students, but may also keep them psychologically oriented towards possible updates, interactions, and opportunities they do not want to miss (Wegmann et al., 2017).

During threat appraisal, students therefore do not simply judge whether information overload is harmful. They may also evaluate what might be lost if they disengage. Such appraisals may make threat appraisal less coherent and more internally conflicted. More specifically, information overload may be appraised as a depleting threat that is linked to lower energy, which is reflected in social media fatigue, while at the same time being appraised as a possible threat of social or informational loss, which is reflected in fear of missing out. Theoretically, this dual appraisal is important because fear of missing out does not merely represent distress. It is also associated with an approach-oriented motivation to remain informed, visible, and socially connected (Fioravanti et al., 2021; Przybylski et al., 2013). As a result, although fear of missing out may arise alongside information overload, its function appears to differ from that of social media fatigue. Social media fatigue is more closely related to stepping back from continued engagement, whereas fear of missing out is more closely related to monitoring and checking. That distinction may help explain why fear of missing out was associated with information overload, yet was not clearly associated with reflective smartphone disengagement.

Internal conflict may leave students with a sense of digital powerlessness. Even when they feel tired from excessive information, they may still worry about missing important developments (Alabri, 2022). While constant notifications are linked to greater social media fatigue, fear of missing out may sustain vigilance rather than support disconnection. Previous research has shown that fear of missing out is more consistently associated with social media use, problematic social networking site use, and compulsive tendencies to stay connected than with deliberate efforts to disengage from digital media (Fioravanti et al., 2021; Wegmann et al., 2017). In addition, recent evidence suggests that fear of missing out is associated with weaker inhibitory control in social media-related contexts, especially when individuals are exposed to salient platform cues (Xu & Tian, 2023). Accordingly, fear of missing out may be particularly responsive to cues that invite checking and updating, rather than to reflective decisions about regulating smartphone use. The feeling that one “should not miss the development” has become part of everyday digital life (Argan et al., 2018). Because many students lack effective strategies for coping with information overload, they may be unable either to manage information efficiently or to disengage comfortably (Tanhan et al., 2022).

### From information overload to disengagement

The findings suggest that reflective smartphone disengagement may function as a protective behaviour when students feel burdened by digital information. Social media fatigue appears to be more closely aligned with reflective smartphone disengagement because both involve a recognition that continued engagement is psychologically costly and that stepping back may be beneficial. Prior research has likewise linked social media fatigue to withdrawal intentions and reduced engagement in social media environments (Luqman et al., 2017). In the present study, the positive association between social media fatigue and reflective smartphone disengagement indicates that, when students experience cognitive and emotional exhaustion from excessive digital input, disengagement may serve as a practical means of relieving strain, limiting further stimulation, and restoring a sense of psychological control.

Once information overload is appraised as burdensome, students may become more inclined to adopt a coping behaviour that reduces continued exposure to the stimulus. Within this process, social media fatigue reflects not only a state of depletion, but also a psychological condition under which disengagement becomes more likely. Reflective smartphone disengagement therefore appears to involve more than a passive reduction in smartphone use. Rather, it may represent a deliberate and self-protective effort to manage the emotional and attentional costs associated with ongoing digital demands.

Fear of missing out, by contrast, was not significantly associated with reflective smartphone disengagement. Although this result did not support the relevant hypothesis, it remains theoretically informative. Fear of missing out is generally understood as a state that encourages continued orientation towards social information, frequent checking, and the maintenance of connection (Fioravanti et al., 2021; Przybylski et al., 2013). Reflective smartphone disengagement, however, involves deliberately restricting smartphone use in order to regulate one’s behaviour (Matthes et al., 2022). A student who is tired of constant information flows may choose to step back from smartphone use, whereas a student experiencing fear of missing out may remain mentally focused on updates, interactions, and possible developments that could be missed. Under these conditions, fear of missing out may sustain attentional vigilance rather than encourage disengagement.

Although information overload may increase fear of missing out, fear of missing out itself may not directly guide people towards or away from reflective smartphone disengagement in a stable manner. One possible reason is that fear of missing out is more strongly tied to monitoring motives than to behavioural self-regulation. In a highly connected environment, people may remain psychologically connected even while reducing smartphone use in specific situations, for example, by checking later, shifting to other devices, or selectively reconnecting when needed (Syvertsen & Enli, 2020). Under such conditions, fear of missing out may persist as an internal concern without becoming a behavioural barrier to reflective smartphone disengagement. Another possible explanation is that the relationship between fear of missing out and reflective smartphone disengagement may be dynamic rather than immediately observable in a cross-sectional model. Recent longitudinal evidence suggests that fear of missing out and reflective smartphone disengagement may negatively predict each other over time, pointing to an ongoing competition between impulsive motivations to stay connected and reflective efforts to regulate smartphone use (Matthes et al., 2023). Such findings imply that fear of missing out may still matter for disengagement, but not necessarily as a straightforward concurrent predictor in a single-wave design. The broader fear of missing out literature also indicates conceptual and operational diversity, suggesting that its behavioural consequences may vary across contexts, platforms, and outcome variables (Tandon et al., 2021).

### The important role of self-efficacy: enhancing digital literacy

The self-efficacy analysis highlights important boundary conditions for digital disconnection. The empirical findings were contrary to the initial predictions in H8a and H8b: rather than strengthening the positive associations between information overload, social media fatigue and reflective smartphone disengagement, self-efficacy attenuated these relationships. While the original hypotheses were informed by a conventional reading of protective theories, where higher perceived competence is expected to facilitate protective actions, the observed pattern suggests that the functional role of self-efficacy in digital contexts is more nuanced.

A plausible reconciliation of these counter-intuitive findings lies in distinguishing between two orientations of strategy: proactive cognitive management and behavioural avoidance. Reflective smartphone disengagement may function differently depending on context; although it can represent deliberate self-protection, under conditions of overload and fatigue it may also resemble withdrawal from demands. Importantly, lower self-efficacy is commonly associated with a heightened reliance on avoidance-oriented coping mechanisms (De Castella et al., 2018). Consequently, when confronted with overwhelming information overload and social media fatigue, individuals with limited self-efficacy exhibit a stronger inclination towards complete disengagement as a means of eliminating the perceived threat. This mechanism explains why the positive associations between information overload, social media fatigue and disengagement is notably more pronounced among those with lower self-efficacy. These findings corroborate prior evidence that personal beliefs are closely intertwined with the selection of specific protective strategies (Gori et al., 2023), and further confirm that stronger internal coping skills are associated with a reduced dependence on avoidance-based approaches (Bhati & Sethy, 2022).

Protection motivation theory often posit that stronger self-efficacy aligns with stronger protective responses (Maddux & Rogers, 1983; Rogers, 1975). However, the present findings point to a more context-sensitive reading within digital environments, suggesting that reflective smartphone disengagement represents only one possible protective option, rather than the universally preferred response. Students with higher self-efficacy may possess a broader coping repertoire, enabling them to employ proactive strategies, such as information filtering, boundary setting, and selective management, rather than relying primarily on complete disengagement (Dienlin & Johannes, 2020; V. T. Nguyen, 2022; M. H. Nguyen et al., 2020). In digitally demanding settings, disengagement may at times be more closely related to limited coping confidence or to more avoidance-oriented forms of coping (Biggs et al., 2017). Fundamentally, reflective smartphone disengagement entails proactively finding a sustainable balance between digital connection and disconnection to safeguard personal well-being. Individuals with higher self-efficacy exhibit greater mastery over their digital environments; this enhanced control allows them to effectively regulate their device usage (Matthes et al., 2022), thereby mitigating the necessity to resort to forced disconnection. Seen in this light, self-efficacy relates not only to whether individuals seek protection but, more critically, to the form that such protection takes.

Therefore, executing appropriate protective actions emerges as a multifaceted skill in contemporary digital societies (Tinmaz et al., 2022). This competency extends beyond basic information management and is strongly associated with independent thinking and decision-making (Pérez-Escoda et al., 2024). Consequently, educational initiatives may benefit from encouraging proactive and efficient information-processing strategies rather than focusing solely on technological avoidance. Because self-efficacy underpins the ability to implement such proactive strategies, fostering young people’s self-efficacy remains an important goal. This involves strengthening their capability to comprehend, evaluate, and filter information. Such cognitive abilities are associated with greater confidence in complex digital environments (Rohde et al., 2023), guiding the selection of optimal protective behaviours.

Regarding fear of missing out, the moderating effect of self-efficacy was not significant. Unlike information overload and social media fatigue, which arise from excessive external demands, fear of missing out may be less sensitive to self-efficacy beliefs (Tanhan et al., 2022), as it is driven more by social-comparison (Festinger, 1954; Neumann, 2020) and reward-sensitivity mechanisms (Tandon et al., 2021) than by perceived coping ability.

### Implication

#### Theoretical implication

The findings place information overload within the logic of protection motivation theory. Previous research has often discussed information overload in close connection with strain, digital stress, or other negative psychological outcomes (Hall et al., 2021; Ou et al., 2023; Whelan et al., 2020). Such an approach has been useful, but it can also blur the distinction between the external digital condition and the individual’s subjective evaluation of that condition. Protection motivation theory provides a different lens by separating environmental stimuli from threat appraisal and coping appraisal (Maddux & Rogers, 1983; Rogers, 1975; Weinstein, 1993). Read from this perspective, information overload may be understood less as stress itself and more as a condition whose meaning depends on how students appraise it. In this respect, the present findings not only echo earlier work on information overload and digital strain (Ou et al., 2023; Ye et al., 2023), but also extend that line of research by suggesting that the association between information overload and reflective smartphone disengagement is not adequately captured by a simple stress-response interpretation.

The results also add nuance to how protection motivation theory may be applied in digital settings. In many classical discussions, threatening conditions are relatively clear, and stronger threat appraisal is usually discussed as being aligned with stronger protective motivation (Balla & Hagger, 2025; Rogers, 1975; Yuniawan et al., 2025). In the present context, however, information overload was associated with both social media fatigue and fear of missing out, and these two states appear to carry different theoretical meanings. Social media fatigue is more readily connected to depletion and the desire to step back, whereas fear of missing out is more closely tied to concern about losing social or informational connections. This pattern suggests that, in digital environments, threat appraisal may be more internally differentiated than is often assumed. Rather than pointing to a single protective orientation, appraisal may involve simultaneous tension between self-protection and continued connection. This interpretation resonates with broader discussions of digital ambivalence, which describe users as navigating the competing demands of availability, participation, and withdrawal (Bayer et al., 2016; Halfmann & Rieger, 2019).

Reflective smartphone disengagement can therefore be interpreted as more than a direct behavioural correlate of information overload. The present findings suggest that it may be better understood as a form of reflective coping situated within a psychologically conflicted digital context. From this viewpoint, disconnection is not simply the opposite of connection, nor is it necessarily an automatic response to overload. Instead, it appears to be embedded in a broader process of digital self-regulation in which tendencies to disengage coexist with motives for monitoring, checking, and staying socially available. This helps enrich the literature on digital behaviour by moving beyond a binary view of connection versus disconnection and by situating reflective smartphone disengagement within the broader negotiation of everyday digital life (Schmuck, 2020).

Given the cross-sectional design, these points should be interpreted as theoretical extensions and interpretive insights based on associations, not as evidence of causal direction or underlying mechanisms. Even so, they offer a more refined account of how students may negotiate the competing pressures of connection and disconnection in everyday digital life.

#### Practical implication

This research provides empirical evidence to reconsider widespread smartphone bans in school environments. Current educational policies and parental interventions often aim to shield students from information overload. These methods typically involve confiscating devices or restricting screen time (Gao et al., 2014). However, mandatory bans are merely associated with short-term reductions in device usage. They rarely address the core issues of digital literacy and self-regulation (Beneito & Vicente-Chirivella, 2022). The current findings indicate that physical disconnection often reflects an avoidance tendency under information overload. Consequently, mandatory bans may block information flow but fail to alleviate the internal anxiety linked to the fear of missing out (Radtke et al., 2022). Therefore, policymakers should recognise the limitations of strictly restrictive policies (Lemahieu et al., 2025). Educational approaches must shift from passive isolation towards active media management.

Interventions should target students experiencing psychological conflict under information overload. Programmes can focus on enhancing their capacity to navigate complex digital environments (Jones et al., 2018). For example, cognitive behavioural therapy can teach students to actively filter irrelevant information using technical tools. This method offers a practical alternative to simply advising a complete cessation of smartphone use (Grace-Farfaglia, 2019). They should guide students to take protective behaviours to avoid threats, more importantly, adopt diverse protective behaviours. Clinical interventions that build self-efficacy frequently correspond with greater cognitive flexibility. This flexibility links closely to positive mental health states. As a result, university students can protect themselves without relying on extreme reflective smartphone disengagement.

### Limitation

This study has several limitations. First, the cross-sectional design reveals associations among variables but constrains causal inferences. Future research could employ longitudinal or experimental designs to examine temporal order and causality. Second, the research only targeted university students. Researchers should exercise caution when generalising these results to other user groups. Third, only gender was included as a control variable. This approach does not rule out the possible influence of other background factors, such as academic level, major, or family socioeconomic context. These unmeasured factors may have introduced additional variance not captured in the present model. Therefore, the findings should be interpreted with caution. Future studies should include a broader range of demographic and contextual variables, where theoretically warranted, to test the robustness of the present results. Last, this study positions the protective measure specifically as disconnecting from the mobile phone. However, individuals might still access social media platforms through desktop computers or tablets. Consequently, reflective smartphone disengagement might not necessarily represent an absolute reduction in social media use. This specific behaviour might simply reflect an alteration in the timing and format of digital engagement. Future studies should focus on different disconnection strategies and information management techniques. Researchers need to investigate how individuals use multiple smart devices across broader information environments.

## Conclusion

These findings further suggest that maintaining well-being in the digital age depends on possessing proactive information regulation strategies. Students who skilfully employ such strategies can embrace complex information with calm, whereas those who lack them risk descending into anxiety, and only take reflective smartphone disengagement as a protective behaviour. Therefore, future research should go beyond mere description of phenomena and instead focus on enabling individuals to develop flexible and sustainable digital resilience, which will be crucial for users to maintain mental well-being and cognitive autonomy in the digital world.

## Data Availability

The data that support the findings of this study are available from the corresponding author upon reasonable request.” to: “The data that support the findings of this study are available from the author Yiwei Wu (wu_yiwei@foxmail.com) upon reasonable request.
